# Observability Analysis of a Matrix Kalman Filter-Based Navigation System Using Visual/Inertial/Magnetic Sensors

**DOI:** 10.3390/s120708877

**Published:** 2012-06-27

**Authors:** Guohu Feng, Wenqi Wu, Jinling Wang

**Affiliations:** 1 The College of Mechatronics and Automation, National University of Defense Technology, Changsha 410073, Hunan, China; E-Mail: wenqiwu_lit@hotmail.com; 2 School of Surveying and Spatial Information Systems, University of New South Wales, Sydney, NSW 2052, Australia; E-Mail: jinling.wang@unsw.edu.au

**Keywords:** matrix Kalman filter, Lie derivatives, observability of nonlinear systems, navigation, vision, inertial measurement unit

## Abstract

A matrix Kalman filter (MKF) has been implemented for an integrated navigation system using visual/inertial/magnetic sensors. The MKF rearranges the original nonlinear process model in a pseudo-linear process model. We employ the observability rank criterion based on Lie derivatives to verify the conditions under which the nonlinear system is observable. It has been proved that such observability conditions are: (a) at least one degree of rotational freedom is excited, and (b) at least two linearly independent horizontal lines and one vertical line are observed. Experimental results have validated the correctness of these observability conditions.

## 1. Introduction

Inertial navigation systems (INS) have been widely used in many systems, such as ground vehicles, airplanes, helicopters, robotic systems, *etc.* However, INS drift will lead to exponential growth of errors in the navigation solutions due to the double integration of acceleration signals within inertial navigation computations. Therefore, to overcome such a problem, it is a very common practice to integrate INS with other sensors, which can calibrate the inertial sensor errors. In most outdoor applications, a Kalman filter estimator can be used for optimally combining both IMU and GPS measurements [1]. However, an indoor navigation system cannot use GPS since its signals are not available.

An alternative approach to calibrate INS errors is via the use of other sensors, such as cameras and magnetic sensors. Combining these two sensors to form a vision-aided inertial navigation system (V-INS) has recently become a popular topic of research [2]. By sensing the Earth's magnetic field a magnetic sensor can provide a drift-free heading estimate. Accurate 3-D orientation estimates of a rigid body by inertial/magnetic sensing were exploited in [3], where the aiding sensors (accelerometer and magnetic sensor) helped mitigate low-frequency gyro drift errors, while, in turn, the signals from the aiding sensors, which are prone to relatively high-frequency errors, are smoothed using gyro data. They are all based on the concept of vector matching, which requires, in principle, the measurements of constant reference vectors (e.g., gravity and the Earth's magnetic field) [4].

In this paper, we present a matrix Kalman filter (MKF) in which the estimate of the state matrix is expressed in terms of the matrix parameters of the original plant. The MKF has the statistical properties of the ordinary EKF, while retaining the advantages of a compact matrix notation by expressing the estimated matrix in terms of the original plant parameters [5].

The major contribution of this paper is to elucidate under which conditions a MKF-based nonlinear system for indoor navigation using visual/inertial/magnetic sensors is observable; in other words, the conditions when sufficient information is available for estimating a state matrix that contains, in the present case, the body attitude matrix, the gyro bias vector, relative velocity vector, the dual part of landmark and the magnetic variation superimposed to the magnetic reference vector. For the purpose of orientation determination, an accurately known homogeneous magnetic field in the environment is needed. Magnetic homogeneity is difficult to achieve, especially indoors, due to the presence of iron construction materials in floors, walls and ceilings, or to interferences from various types of equipment. In order to compensate for magnetic variations, a first-order Gauss-Markov vector random process is chosen to model the magnetic variation. To the best of our knowledge, there has been no such observability analysis so far for the integrated navigation systems in 3-D. We have extended the current work for the observability analysis for an orientation system described in [6], to the 3-D navigation systems based on inertial/visual/magnetic sensors.

## 2. Sensor Modeling

### 2.1. Inertial Sensor

**Figure 1.**
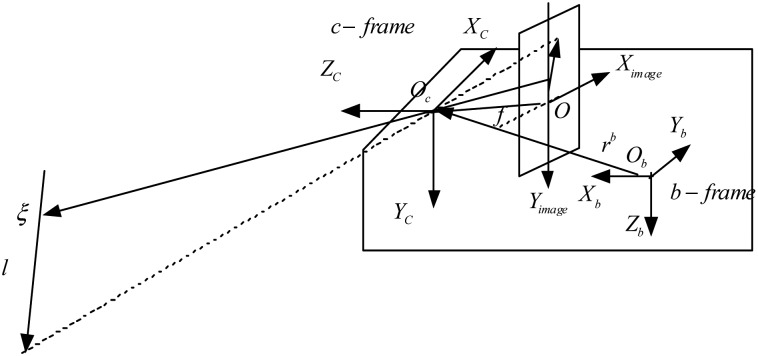
Geometry of visual/inertial/magnetic sensor based navigation.

Without loss of generality, the navigation reference frame is selected as the local-level frame (North-East-Down). Denote the navigation frame by *n*, the INS body frame by *b*, the camera frame by *c* and the inertial frame by *i*. Using gyros/accelerometers outputs, the relative velocity *v^n^* and the body attitude matrix ${\text{C}}_{n}^{b}$ satisfy the kinematic equations as [1,7]:

(1) $${\dot{\xi }}^{n}=-{\text{v}}^{n}$$

(2) $${\dot{v}}^{n}={\text{C}}_{\text{b}}^{n}\left({\text{f}}^{b}-b_{a}\right)+{\text{g}}^{n}$$

(3) $${\dot{\text{C}}}_{n}^{\text{b}}=-\left[\omega_{nb}^{b}\times \right]C_{n}^{b},\omega_{nb}^{b}=\omega_{ib}^{b}-b_{g}$$

(4) $${\dot{b}}_{g}={\text{n}}_{\omega g}$$

where **ξ***^n^* is an arbitrary point on the observed line, **r***^b^* is the lever arm from the IMU to the camera, as shown in Figure 1. **b***_a_* and **b***_g_* are 3 × 1 vectors that describe the biases affecting the accelerometer and gyro measurements, respectively. **b***_a_* can be compensated on time scales up to few hours, using the procedure described in [8]. **b***_g_* are modeled as random walk processes, driven by the white Gaussian noise vectors **n***_ωg_*. $\left[\omega_{nb}^{b}\times \right]$ is the skew symmetric matrix of $\omega_{nb}^{b}$. In the context of MEMS sensors, the component in the gyro output due to the Earth's rotation can be neglected as compared to the sensor errors.

### 2.2. Visual Sensor

The line point is taken as line representation, which is defined as the intersection of a line feature with a line passing through the image origin that is perpendicular to the line feature. The line point is unique for all lines except the lines passing through the origin. The line point is calculated by Goddard as [9]:

(5) $$x_{lp}=\frac{fm_{x}^{c}m_{z}^{c}}{m_{x}^{c2}+m_{y}^{c2}},y_{lp}=\frac{fm_{y}^{c}m_{z}^{c}}{m_{x}^{c2}+m_{y}^{c2}}$$

Line features are represented by quaternion. **Î** = **l** + ε**m**, where **l** is the unit direction vector of the observed line and **m** is related to the position by **m** = **p** × **1**. In the *n*-frame:

(6) $${\text{m}}^{n}=\xi^{n}\times {\text{l}}^{n}$$

while in the *c*-frame:

(7) $$\begin{array}{cc} {\text{m}}^{c} & =\left({\text{C}}_{n}^{c}\left(\xi^{n}-{\text{C}}_{n}^{c}{\text{r}}^{b}\right)\right)\times \left({\text{C}}_{n}^{c}{\text{l}}^{n}\right)={\text{C}}_{n}^{c}\left(\left(\xi^{n}-{\text{C}}_{n}^{c}{\text{r}}^{b}\right)\times {\text{l}}^{n}\right)\\ & ={\text{C}}_{n}^{c}{\text{m}}^{n}-{\text{C}}_{n}^{c}{\text{r}}^{b}\times {\text{l}}^{n}\approx {\text{C}}_{n}^{c}{\text{m}}^{n} \end{array}$$

where **r***^b^* can be ignored when the IMU and the camera are mounted closely together.

If relative position **ξ***^n^* is orthogonal to **l***^n^*, that is, **ξ***^n^*·**l***^n^* = 0, then we can obtain:

(8) $$\left\|{\text{m}}^{n}\right\|=\left\|\xi^{n}\right\|\left\|{\text{l}}^{n}\right\|=\left\|\xi^{n}\right\|$$

That is to say, the norm of **m***^n^* is the minimum distance from the vehicle to the observed line. Using Equation (1), we obtain:

(9) $${\dot{m}}^{n}=\xi^{n}\times {\text{l}}^{n}=-{\text{v}}^{n}\times {\text{l}}^{n}=[{\text{l}}^{n}\times ]{\text{v}}^{n}$$

For simplification, only vertical lines ${\text{l}}_{v}^{n}$ and horizontal lines ${\text{l}}_{h}^{n}$ are chosen as landmarks. According to Equation (5), we obtain:

(10) $${\text{m}}^{c}={\left[\begin{array}{ccc} m_{x} & m_{y} & m_{z} \end{array}\right]}^{T}={\left[\begin{array}{ccc} \frac{fx_{lp}m_{z}}{{x_{lp}}^{2}+{y_{lp}}^{2}} & \frac{fy_{lp}m_{z}}{{x_{lp}}^{2}+{y_{lp}}^{2}} & m_{2} \end{array}\right]}^{T}$$

A monocular camera is not enough to calculate *m_z_* due to its inherent limitation of depth information deficiency. In order to solve this problem, we can use stereo cameras or a monocular camera with height information to obtain *m_z_*.

### 2.3. Magnetic Sensor

A first-order Gauss-Markov vector random process with statistically independent components is chosen to model magnetic variations as follows [10]:

(11) $${\dot{b}}_{h}=-\alpha b_{h}+n_{\omega h}$$

where α is a positive constant, and **n***_ωh_* is white Gaussian noise.

## 3. MKF Algorithm

### 3.1. Process Model

The evolving state is described as follows:

(12) $${\text{X}}_{k}=\left[\begin{array}{cccccc} {\text{C}}_{nk}^{\text{b}} & {\text{b}}_{gk} & {\text{v}}_{k}^{n} & {\text{m}}_{vk}^{n} & {\text{m}}_{hk}^{n} & b_{hk} \end{array}\right]$$

where **m***^n^* is the relative distance of the vehicle with respect to the observed line, **v***^n^* is the relative velocity.

The relative position of the vehicle with respect to the observed line, **ξ***^n^*, can be calculated as follows:

1. Calculate the point of intersection of the observed lines. According to Equation (6), we obtain:
  (13) $$\xi_{i}^{n}=-{\left({\left[{\text{l}}_{v}^{n}\times \right]}^{2}+{\left[{\text{l}}_{h}^{n}\times \right]}^{2}\right)}^{-1}\left(\left[{\text{l}}_{v}^{n}\times \right]{\text{m}}_{v}^{n}+\left[{\text{l}}_{h}^{n}\times \right]{\text{m}}_{h}^{n}\right)$$
2. Calculate the translation along the observed line direction from the intersection to the vertical point. According to Equation (8), we obtain:
  (14) $$\begin{array}{c} \left\|\xi_{⊥v}^{n}\right\|=\left\|\xi_{i}^{n}+\rho_{v}{\text{l}}_{v}^{n}\right\|=\left\|{\text{m}}_{v}^{n}\right\|\\ \left\|\xi_{⊥h}^{n}\right\|=\left\|\xi_{i}^{n}+\rho_{h}{\text{l}}_{h}^{n}\right\|=\left\|{\text{m}}_{h}^{n}\right\| \end{array}$$
  Because the unique of perpendicular point, the translation along the observed line direction, *ρ*, is also uniqueness. From Equation (14), we obtain:
  (15) $$\begin{array}{cc} \rho_{v}=-\xi_{i}^{nT}l_{v}^{n} & \rho_{h}=-\xi_{i}^{nT}l_{h}^{n} \end{array}$$
3. Calculate the relative position:
  (16) $$\begin{array}{c} \xi_{⊥v}^{n}=\xi_{i}^{n}+\rho_{v}{\text{l}}_{v}^{n}=\xi_{i}^{n}-\left(\xi_{i}^{nT}{\text{l}}_{v}^{n}\right){\text{l}}_{v}^{n}\\ \xi_{⊥h}^{n}=\xi_{i}^{n}+\rho_{h}{\text{l}}_{h}^{n}=\xi_{i}^{n}-\left(\xi_{i}^{nT}{\text{l}}_{h}^{n}\right){\text{l}}_{h}^{n} \end{array}$$

The discretization of Equation (3) gives the following equation [11]:

(17) $${\text{C}}_{nk+1}^{\text{b}}=\Phi_{k}{\text{C}}_{nk}^{b}+[b_{g}\times ]{\text{C}}_{nk}^{b}\Delta t+{\text{w}}_{k}$$

where Φ*_k_* is the transition matrix from time *tk* to time *tk*_+1_ that corresponds to $-[\omega_{ib}^{b}\times ]$. We assume that $\omega_{ib}^{b}$ is piece wisely constant in the tiny time intervals *Δt* = *tk*_+1_ − *tk*, and then, Φ*_k_* in Equation (17) can be approximated as:

(18) $$\Phi_{k}\approx \exp^{-[\omega_{ib}^{b}\times ]\Delta t}$$

Furthermore, **w***_k_* can be written as:

(19) $${\text{w}}_{k}[{\text{n}}_{g}\times ]{\text{C}}_{nk}^{b}\Delta t+h.o.t$$

where *h.o.t* denotes the terms of the second order *Δt*^2^ and higher. Note that the process noise matrix, **w***_k_*, is state dependent, and that the first-order term is linear in the components of the white Gaussian noise vectors **n***_g_*.

Discretizing Equations (2), (4), (9), (11) produces:

(20) $${\text{b}}_{gk+1}={\text{b}}_{gk}+{\text{n}}_{\omega g}\Delta t$$

(21) $${\text{v}}_{k+1}^{n}={\text{v}}_{k}^{n}+{\text{C}}_{b}^{n}{\text{f}}^{b}\Delta t+{\text{g}}^{n}\Delta t$$

(22) $${\text{m}}_{k+1}^{n}={\text{m}}_{k}^{n}+[{\text{l}}^{n}\times ]{\text{v}}^{n}\Delta t$$

(23) $${\text{b}}_{hk+1}=\exp^{-\alpha \Delta t}{\text{b}}_{hk}+{\text{n}}_{\omega h}\Delta t$$

Combining Equations (17), (20)–(23) together, we obtain the dynamic model as:

(24) $${\text{X}}_{k+1}=\sum_{r=1}^{8}\Theta_{k}^{r}{\text{X}}_{k}\Lambda_{k}^{r}+{\text{B}}_{k}{\text{U}}_{k}\text{E}+{\text{W}}_{k}$$

where the dynamic matrices, $\Theta_{k}^{r}$, $\Lambda_{k}^{r}$, are defined as:

(25) $$\begin{array}{ll} \Theta_{k}^{1}=\Phi_{k} & \Lambda_{k}^{1}={\text{E}}^{11}+{\text{E}}^{22}+{\text{E}}^{23}\\ \Theta_{k}^{2}=[{\text{c}}_{1k}\times ] & \Lambda_{k}^{2}={\text{E}}^{41}\Delta t\\ \Theta_{k}^{3}=[{\text{c}}_{2k}\times ] & \Lambda_{k}^{3}={\text{E}}^{42}\Delta t\\ \Theta_{k}^{4}=[{\text{c}}_{3k}\times ] & \Lambda_{k}^{4}={\text{E}}^{43}\Delta t\\ \Theta_{k}^{5}={\text{I}}_{3} & \Lambda_{k}^{5}={\text{E}}^{44}+{\text{E}}^{55}+{\text{E}}^{66}+{\text{E}}^{77}\\ \Theta_{k}^{6}=[{\text{I}}_{v}^{n}\times ] & \Lambda_{k}^{6}={\text{E}}^{56}\Delta t\\ \Theta_{k}^{7}=[{\text{I}}_{h}^{n}\times ] & \Lambda_{k}^{7}={\text{E}}^{57}\Delta t\\ \Theta_{k}^{8}=\exp^{-\alpha \Delta t}{\text{I}}_{3} & \Lambda_{k}^{8}={\text{E}}^{88} \end{array}$$

where **E***^ij^* denotes a 8 × 8 matrix with 1 at position (*ij*) and 0 elsewhere;

(26) $$\text{B}=[\begin{array}{cc} {\text{C}}_{nk}^{bT} & {\text{I}}_{3} \end{array}],{\text{U}}_{k}=\left[\begin{array}{c} {\text{f}}_{k}^{b}\\ {\text{g}}^{n} \end{array}\right],\text{E}=\left[\begin{array}{cc} {\text{e}}_{5}^{T}\Delta t & 0_{3\times 1} \end{array}\right]$$

and the noise matrix, **W***_k_*, is defined as:

(27) $${\text{W}}_{k}=[\begin{array}{llllll} {\text{w}}_{k} & {\text{n}}_{\omega g}\Delta t & {\text{n}}_{a}\Delta t & {\text{n}}_{c}\Delta t & {\text{n}}_{c}\Delta t & {\text{n}}_{h}\Delta t \end{array}]$$

The process noise covariance matrix is:

(28) $${\text{Q}}_{k}=\left[\begin{array}{cccccc} \mathrm{cov}[{\text{w}}_{k}] & 0_{9\times 3} & 0_{9\times 3} & 0_{9\times 3} & 0_{9\times 3} & 0_{9\times 3}\\ 0_{3\times 9} & \sigma_{\omega g}^{2}\Delta t{\text{I}}_{3} & 0_{3\times 3} & 0_{3\times 3} & 0_{3\times 3} & 0_{3\times 3}\\ 0_{3\times 9} & 0_{3\times 3} & \sigma_{a}^{2}\Delta t{\text{I}}_{3} & 0_{3\times 3} & 0_{3\times 3} & 0_{3\times 3}\\ 0_{3\times 9} & 0_{3\times 3} & 0_{3\times 3} & \sigma_{c}^{2}\Delta t{\text{I}}_{3} & 0_{3\times 3} & 0_{3\times 3}\\ 0_{3\times 9} & 0_{3\times 3} & 0_{3\times 3} & 0_{3\times 3} & \sigma_{c}^{2}\Delta t{\text{I}}_{3} & 0_{3\times 3}\\ 0_{3\times 9} & 0_{3\times 3} & 0_{3\times 3} & 0_{3\times 3} & 0_{3\times 3} & \sigma_{h}^{2}\frac{1-\exp^{-2\alpha \Delta t}}{2\alpha }{\text{I}}_{3} \end{array}\right]$$

where, as defined by [11]:

(29) $$\mathrm{cov}[{\text{w}}_{k}]=\left({\left({\hat{C}}_{nk}^{b}\right)}^{T}⊗{\text{I}}_{3}\right)\text{L}\sigma_{g}^{2}{\text{I}}_{3}{\text{L}}^{T}\left({\hat{C}}_{nk}^{b}⊗{\text{I}}_{3}\right)\Delta t^{2}$$

**L** is the 9 × 3 matrix defined as:

(30) $${\text{L}}^{T}=\left[\begin{array}{ccc} \left[{\text{e}}_{1}\times \right] & \left[{\text{e}}_{2}\times \right] & \left[{\text{e}}_{3}\times \right] \end{array}\right]$$

with **e**_1_ = [1 0 0]*^T^*, **e**_2_ = [0 1 0]*^T^*, and **e**_3_ = [0 0 1]*^T^*.

The process Equation (24) is not linear because in the matrix $\Theta_{k}^{r}$, *r* = 2, 3, 4, are the functions of elements of ${\text{C}}_{nk}^{b}$. One way of overcoming this difficulty is to substitute ${\hat{C}}_{nk/k}^{b}$ for ${\text{C}}_{nk}^{b}$ in the process equation. This substitution yields a pseudo-linear process equation.

### 3.2. Measurement Model

The matrix measurement equation:

(31) $${\text{Y}}_{k+1}=\left[\begin{array}{cc} {\text{m}}_{k+1}^{c} & {\text{m}}_{mk+1} \end{array}\right]=\sum_{s=1}^{2}{\text{H}}_{k+1}^{s}{\text{X}}_{k+1}{\text{G}}_{k+1}^{s}+{\text{V}}_{k+1}$$

where the measurement matrices are:

(32) $$\begin{array}{c} {\text{H}}_{k+1}^{1}={\text{C}}_{b}^{c},{\text{G}}_{k+1}^{1}=\left[\begin{array}{cc} {\text{m}}_{k}^{n} & 0_{3\times 1}\\ 0_{5\times 1} & 0_{5\times 1} \end{array}\right]\\ {\text{H}}_{k+1}^{2}={\text{I}}_{3},{\text{G}}_{k+1}^{2}=\left[\begin{array}{cc} 0_{3\times 1} & \text{h}+b_{h}\\ 0_{5\times 1} & 0_{5\times 1} \end{array}\right] \end{array}$$

and the measurement noise covariance matrix is given as:

(33) $${\text{R}}_{k+1}=\left[\begin{array}{cc} \sigma_{c}^{2}{\text{I}}_{3} & 0_{3\times 3}\\ 0_{3\times 3} & \sigma_{h}^{2}{\text{I}}_{3} \end{array}\right]$$

### 3.3. Update

Putting *m* = 3, *n* = 8, *p* = 3, *q* = 2, *μ*= 8, *v* = 2 into Equations described in [5], we can obtain time update and measurement update equations. However, due to the existence of the input matrix, the time update equation below is not the same as that given in [15]:

(34) $${\hat{X}}_{k+1/k}=\sum_{r=1}^{8}\Theta_{k}^{r}{\hat{X}}_{k/k}\Lambda_{k}^{r}+{\text{B}}_{k}{\text{U}}_{k}\text{E}$$

(35) $$\Phi_{k}=\sum_{r=1}^{8}\left[{\left(\Lambda_{k}^{r}\right)}^{T}⊗\Theta_{k}^{r}\right]$$

(36) $${\text{P}}_{k+1/k}=\Phi_{k}{\text{P}}_{k/k}\Phi_{k}^{T}+{\text{Q}}_{k}$$

Measurement update equations:

(37) $${\hat{\text{Y}}}_{k+1}=\sum_{s=1}^{2}{\text{H}}_{k+1}^{s}{\hat{X}}_{k+1/k}{\text{G}}_{k+1}^{s}-Y_{k+1}$$

(38) $${\text{H}}_{k+1}=\sum_{s=1}^{2}\left[{\left({\text{G}}_{k+1}^{s}\right)}^{T}⊗{\text{H}}_{k+1}^{s}\right]$$

(39) $${\text{S}}_{k+1}={\text{H}}_{k+1}{\text{P}}_{k+1/k}{\text{H}}_{k+1}^{T}+{\text{R}}_{k+1}$$

(40) $${\text{K}}_{k+1}={\text{P}}_{k+1}{\text{H}}_{k+1}^{T}{\text{S}}_{k+1}^{-1}$$

(41) $${\hat{X}}_{k+1/k+1}={\hat{X}}_{k+1/k}-\sum_{j=1}^{8}\sum_{l=1}^{2}{\text{K}}_{k+1}^{jl}{\tilde{Y}}_{k+1}{\text{E}}^{lj}$$

where ⊗ denotes the Kronecker product [12], ${\text{K}}_{k+1}^{jl}$ is a 3 × 3 submatrix of the 24 × 6 matrix **K**_k+1_ defined by:

(42) $${\text{K}}_{k+1}=\left[\begin{array}{cc} {\text{K}}_{k+1}^{11} & {\text{K}}_{k+1}^{12}\\ \vdots & \vdots \\ {\text{K}}_{k+1}^{81} & {\text{K}}_{k+1}^{82} \end{array}\right]$$

and **E***^lj^* is a 2 × 8 matrix with 1 at position (*ij*) and 0 elsewhere:

(43) $$P_{k+1/k+1}=(I_{24}-K\text{H})P_{k+1/k}{\left(I_{24}-K\text{H}\right)}^{T}+KRK^{T}$$

### 3.4. Orthogonalization

The Kalman filter is not designed to preserve any relationship among the components of the estimated state matrix ${\hat{C}}_{nk/k}^{b}$. It would not preserve the orthogonality. Iterative brute-force orthogonalization is presented for its low computation load [11]. The iterative brute-force procedure is as follows [13]:

(44) $${\text{C}}_{n}^{b*}={\hat{C}}_{n}^{b}\left(1.5{\text{I}}_{3}-0.5{\hat{C}}_{n}^{bT}{\hat{C}}_{n}^{b}\right)$$

Although this algorithm is suboptimal, it is much simpler than the optimal algorithm given by Equation (45). When applied recursively, this algorithm produces a sequence of estimates that converge to the optimal solution of Equation (45) [14]. Moreover, as evidenced by simulations, orthogonality can be usually reached in all practical applications after just one or two iterations of Equation (44):

(45) $${\text{C}}_{n}^{b*}={\left({\hat{C}}_{n}^{b}{\hat{C}}_{n}^{bT}\right)}^{-\frac{1}{2}}{\hat{C}}_{n}^{b}$$

## 4. Observability Analysis

A system is observable if its states at a certain time instant can be uniquely determined given a finite sequence of its outputs [15]. Intuitively, observability means that the measurements of an observable system can provide sufficient geometric information for estimating its states. In contrast, the state vector of an unobservable system cannot be recovered regardless of the duration of the estimation process.

In this paper, we study the observability of the nonlinear system describing the visual/inertial/magnetic sensor based navigation process. The system state vector is chosen as follows:

(46) $$\text{x}(t)={\left[\begin{array}{cccccc} {\text{c}}^{T} & {\text{b}}_{g}^{T} & {\text{v}}^{nT} & {\text{m}}_{v}^{nT} & {\text{m}}_{h}^{nT} & {\text{b}}_{n}^{T} \end{array}\right]}^{T}$$

where **c** is defined as:

(47) $$\begin{array}{cc} \text{c}=\left[\begin{array}{c} {\text{c}}_{1}\\ {\text{c}}_{2}\\ {\text{c}}_{3} \end{array}\right], & {\text{C}}_{n}^{b}=[\begin{array}{ccc} {\text{c}}_{1} & {\text{c}}_{2} & {\text{c}}_{3} \end{array}] \end{array}$$

Equation (3) can be rewritten as:

(48) $$\dot{\text{c}}=\Xi \left({\text{b}}_{g}\right)\text{c}-\Xi \left(\omega_{i\text{b}}^{b}\right)\text{c}=\Xi \left(b_{g}\right)c+\text{T}\omega_{ib}^{b}$$

For arbitrary 3 × 1 vector **p**, we define:

(49) $$\Xi (\text{p})=\left[\begin{array}{ccc} \left[\text{p}\times \right] & 0_{3\times 3} & 0_{3\times 3}\\ 0_{3\times 3} & \left[\text{p}\times \right] & 0_{3\times 3}\\ 0_{3\times 3} & 0_{3\times 3} & \left[\text{p}\times \right] \end{array}\right],\text{T}=\left[\begin{array}{c} \left[{\text{c}}_{1}\times \right]\\ \left[{\text{c}}_{2}\times \right]\\ \left[{\text{c}}_{3}\times \right] \end{array}\right]$$

Then, we rearrange the nonlinear kinematic Equations (2), (4), (9), (11), (48) in a pseudo-linear format for computing the Lie derivatives:

(50) $$\left[\begin{array}{c} \dot{\text{c}}\\ {\dot{\text{b}}}_{\text{g}}\\ {\dot{\text{v}}}_{g}\\ {\dot{m}}_{\text{v}}^{n}\\ {\dot{m}}_{h}^{n}\\ {\dot{\text{b}}}_{h} \end{array}\right]=\underset{{\text{f}}_{0}}{\underset{︸}{\left[\begin{array}{c} \Xi (b_{g})c\\ 0_{3\times 1}\\ g^{n}\\ \left[{\text{I}}_{v}^{n}\times \right]v^{n}\\ \left[{\text{I}}_{h}^{n}\times \right]v^{n}\\ -\alpha b_{h} \end{array}\right]}}+\underset{\underline{{\text{f}}_{1}}}{\underset{︸}{\left[\begin{array}{c} \text{T}\\ 0_{3\times 3}\\ 0_{3\times 3}\\ 0_{3\times 3}\\ 0_{3\times 3}\\ 0_{3\times 3} \end{array}\right]}}\omega_{ib}^{b}+\underset{\underline{{\text{f}}_{2}}}{\underset{︸}{\left[\begin{array}{c} 0_{3\times 3}\\ 0_{3\times 3}\\ {\text{C}}_{b}^{n}\\ 0_{3\times 3}\\ 0_{3\times 3}\\ 0_{3\times 3} \end{array}\right]}}{\text{f}}^{b}$$

where $\omega_{ib}^{b}$ and **f***^b^* are the gyro and accelerometer measurements.

Also, note that f_0_ is a 24 × 1 vector, while f̠_1_ and f̠_2_ are both compact representations of three vectors of dimension 24 × 1, *i.e.*,

(51) $$\underline{{\text{f}}_{1}}\omega_{ib}^{b}={\text{f}}_{11}\omega_{x}+{\text{f}}_{12}\omega_{y}+{\text{f}}_{13}\omega_{z}$$

where, for *i* = 1,2,3, f_l_*_i_* denotes the *i* th column vector comprising f̠_1_ and *ω_x_*, *ω_y_*, and *ω_z_* are the scalar components of the rotational velocity vector.

The measurement functions are as follows:

(52) $${\text{h}}^{m}={\text{h}}_{1}^{*}(\text{x})={\text{C}}_{n}^{b}(\text{h}+{\text{b}}_{\text{h}})=\Psi (h+b_{h})\text{c}$$

(53) $${\text{m}}_{n}^{\text{c}}={\text{h}}_{2}^{*}(\text{x})={\text{C}}_{b}^{c}{\text{C}}_{n}^{b}{\text{m}}_{v}^{n}={\text{C}}_{b}^{c}\Psi ({\text{m}}_{v}^{n})c$$

(54) $${\text{m}}_{\text{h}}^{\text{c}}=h_{3}^{*}(\text{x})={\text{C}}_{b}^{c}{\text{C}}_{n}^{b}{\text{m}}_{h}^{n}={\text{C}}_{b}^{c}\Psi ({\text{m}}_{h}^{n})c$$

where:

(55) $$\begin{array}{cc} {\text{C}}_{n}^{b}\text{p}=\Psi (\text{p})\text{c}, & \text{p}={\left[\begin{array}{ccc} p_{1} & p_{2} & p_{3} \end{array}\right]}^{T} \end{array}$$

(56) $$\Psi (\text{p})=[\begin{array}{ccc} p_{1}{\text{I}}_{3} & p_{2}{\text{I}}_{3} & p_{3}{\text{I}}_{3} \end{array}]$$

Furthermore, we enforce the orthogonal constraints by employing the following additional measurement equations:

(57) $$h_{4}^{*}(x)=\left[\begin{array}{c} {c_{1}}^{T}c_{1}\\ {c_{2}}^{T}c_{2}\\ {c_{3}}^{T}c_{3}\\ {c_{11}}^{2}+{c_{12}}^{2}+{c_{13}}^{2}\\ {c_{21}}^{2}+{c_{22}}^{2}+{c_{23}}^{2}\\ {c_{31}}^{2}+{c_{32}}^{2}+{c_{33}}^{2} \end{array}\right]-\left[\begin{array}{c} 1\\ 1\\ 1\\ 1\\ 1\\ 1 \end{array}\right]=0$$

It suffices to show that a subset of the rows of the observability matrix O (*cf.* Equation (75)) are linearly independent. In the remainder of this section, we will prove that the system described by Equations (50)–(57) is observable by computing among the candidate the zeroth- and first- order Lie derivatives of ${\text{h}}_{1}^{*}$, ${\text{h}}_{2}^{*}$, ${\text{h}}_{3}^{*}$, and ${\text{h}}_{4}^{*}$, the ones whose gradients ensure that the observability matrix O is full rank.

- The zeroth-Order Lie Derivatives $\left({𝔏}^{0}{\text{h}}_{1}^{*},{𝔏}^{0}{\text{h}}_{2}^{*},{𝔏}^{0}{\text{h}}_{3}^{*},{𝔏}^{0}{\text{h}}_{4}^{*}\right)$: By definition, the zeroth-order Lie derivative of a function is the function itself, *i.e.*,
  (58) $${𝔏}^{0}{\text{h}}_{1}^{*}={\text{h}}_{1}^{*}={\text{C}}_{n}^{b}(\text{h}+{\text{b}}_{h})=\Psi (h+b_{h})\text{c}$$
  (59) $${𝔏}^{0}{\text{h}}_{2}^{*}={\text{h}}_{2}^{*}={\text{C}}_{b}^{\text{c}}{\text{C}}_{n}^{b}{\text{m}}_{v}^{n}={\text{C}}_{b}^{c}\Psi ({\text{m}}_{v}^{n})\text{c}$$
  (60) $${𝔏}^{0}{\text{h}}_{3}^{*}={\text{h}}_{3}^{*}={\text{C}}_{b}^{\text{c}}{\text{C}}_{n}^{b}{\text{m}}_{h}^{n}={\text{C}}_{b}^{c}\Psi ({\text{m}}_{h}^{n})\text{c}$$
  (61) $${𝔏}^{0}{\text{h}}_{4}^{*}={\text{h}}_{4}^{*}=\left[\begin{array}{c} {{\text{c}}_{1}}^{T}{\text{c}}_{1}\\ {{\text{c}}_{2}}^{T}{\text{c}}_{2}\\ {{\text{c}}_{3}}^{T}{\text{c}}_{3}\\ {c_{11}}^{2}+{c_{12}}^{2}+{c_{13}}^{2}\\ {c_{21}}^{2}+{c_{22}}^{2}+{c_{23}}^{2}\\ {c_{31}}^{2}+{c_{32}}^{2}+{c_{33}}^{2} \end{array}\right]-\left[\begin{array}{c} 1\\ 1\\ 1\\ 1\\ 1\\ 1 \end{array}\right]$$
  Therefore, the gradients of the zeroth-order Lie derivatives are exactly the same as the Jacobians of the corresponding measurement functions:
  (62) $$\nabla {𝔏}^{0}{\text{h}}_{1}^{*}=\left[\begin{array}{ccc} \Psi (\text{h}+{\text{b}}_{h}) & 0_{3\times 12} & {\text{C}}_{n}^{b} \end{array}\right]$$
  (63) $$\nabla {𝔏}^{0}h_{2}^{*}=\left[\begin{array}{cccc} C_{b}^{c}\Psi (m_{v}^{n}) & 0_{3\times 6} & C_{b}^{c}C_{n}^{b} & 0_{3\times 6} \end{array}\right]$$
  (64) $$\nabla {𝔏}^{0}{\text{h}}_{3}^{*}=\left[\begin{array}{cccc} {\text{C}}_{b}^{c}\Psi ({\text{m}}_{h}^{n}) & 0_{3\times 9} & {\text{C}}_{b}^{c}{\text{C}}_{n}^{b} & 0_{3\times 3} \end{array}\right]$$
  (65) $$\nabla {𝔏}^{0}{\text{h}}_{4}^{*}=[\begin{array}{cc} 2\text{M} & 0_{6\times 15} \end{array}]$$
  where:
  (66) $$\text{M}=\left[\begin{array}{ccccccccc} c_{11} & c_{21} & c_{31} & 0 & 0 & 0 & 0 & 0 & 0\\ 0 & 0 & 0 & c_{12} & c_{22} & c_{32} & 0 & 0 & 0\\ 0 & 0 & 0 & 0 & 0 & 0 & c_{13} & c_{23} & c_{33}\\ c_{11} & 0 & 0 & c_{12} & 0 & 0 & c_{13} & 0 & 0\\ 0 & c_{21} & 0 & 0 & c_{22} & 0 & 0 & c_{23} & 0\\ 0 & 0 & c_{31} & 0 & 0 & c_{32} & 0 & 0 & c_{33} \end{array}\right]$$
- The first-Order Lie Derivatives $\left(\nabla {𝔏}_{{\text{f}}_{0}}^{1}{\text{h}}_{2}^{*},\nabla {𝔏}_{{\text{f}}_{0}}^{1}{\text{h}}_{3}^{*},\nabla {𝔏}_{\underline{{\text{f}}_{1}}}^{1}{\text{h}}_{1}^{*},\right)$: The first-order Lie derivatives of $h_{2}^{*}$ and ${\text{h}}_{3}^{*}$ with respect to f_0_ are computed as:
  (67) $$\begin{array}{c} \nabla {𝔏}_{{\text{f}}_{0}}^{1}{\text{h}}_{2}^{*}=\nabla {𝔏}^{0}{\text{h}}_{2}^{*}\cdot {\text{f}}_{0}={\text{C}}_{b}^{c}\Psi \left({\text{m}}_{v}^{n}\right)\Xi \left({\text{b}}_{g}\right)c+{\text{C}}_{b}^{c}{\text{C}}_{n}^{b}\left[{\text{l}}_{\text{v}}^{n}\times \right]v^{n}\\ ={\text{C}}_{b}^{c}\left[b_{g}\times \right]{\text{C}}_{n}^{b}{\text{m}}_{v}^{n}+{\text{C}}_{b}^{c}{\text{C}}_{n}^{b}\left[{\text{l}}_{v}^{n}\times \right]v^{n} \end{array}$$
  (68) $$\nabla {𝔏}_{{\text{f}}_{0}}^{1}{\text{h}}_{3}^{*}=\nabla {𝔏}^{0}{\text{h}}_{3}^{*}\cdot {\text{f}}_{0}={\text{C}}_{b}^{c}\left[b_{g}\times \right]{\text{C}}_{n}^{b}{\text{m}}_{h}^{n}+{\text{C}}_{b}^{c}{\text{C}}_{n}^{b}\left[{\text{l}}_{h}^{n}\times \right]{\text{v}}^{n}$$

Equation (67) is proved in Appendix A, while their gradients are given by:

(69) $$\nabla {𝔏}_{{\text{f}}_{0}}^{1}{\text{h}}_{2}^{*}=\left[\begin{array}{ccccc} {\text{X}}_{1} & -{\text{C}}_{b}^{c}\left[{\text{C}}_{n}^{b}{\text{m}}_{v}^{n}\times \right] & {\text{C}}_{b}^{c}{\text{C}}_{n}^{b}\left[{\text{l}}_{v}^{n}\times \right] & {\text{C}}_{b}^{c}\left[b_{g}\times \right]{\text{C}}_{n}^{b} & 0_{3\times 6} \end{array}\right]$$

(70) $$\nabla {𝔏}_{{\text{f}}_{0}}^{1}{\text{h}}_{3}^{*}=\left[\begin{array}{cccccc} {\text{X}}_{1} & -{\text{C}}_{b}^{c}\left[{\text{C}}_{n}^{b}{\text{m}}_{h}^{n}\times \right] & {\text{C}}_{b}^{c}{\text{C}}_{n}^{b}\left[{\text{l}}_{h}^{n}\times \right] & 0_{3\times 3} & {\text{C}}_{b}^{c}\left[b_{g}\times \right]{\text{C}}_{n}^{b} & 0_{3\times 3} \end{array}\right]$$

In these expressions, X_1_ and X_2_ are both 3 × 9 matrices and regardless of their values, they will be eliminated from the following derivations; hence, they need not be computed explicitly.

The next first-order Lie derivative of interest is that of ${\text{h}}_{1}^{*}$ with respect to f̠_1_, *i.e.*, $\nabla {𝔏}_{\underline{{\text{f}}_{1}}}^{1}{\text{h}}_{1}^{*}$. At this point, it is important to note that f̠_1_ as defined in Equation (50) is a compact representation of three column vectors. Similarly, we can also write the resultant Lie derivative in a compact form (*i.e.*, a 3 × 3 matrix):

(71) $$\nabla {𝔏}_{\underline{{\text{f}}_{1}}}^{1}h_{1}^{*}=\nabla {𝔏}^{0}h_{1}^{*}\cdot \underline{{\text{f}}_{1}}=\Psi (h+b_{h})\text{T}$$

Stacking the gradients of the three columns together gives:

(72) $$\nabla {𝔏}_{\underline{{\text{f}}_{1}}}^{1}{\text{h}}_{1}^{*}=[\begin{array}{ccc} \Gamma & 0_{9\times 12} & \gamma \end{array}]$$

where the matrices:

(73) $$\Gamma =\left[\begin{array}{c} \Gamma_{1}\\ \Gamma_{2}\\ \Gamma_{3} \end{array}\right],\gamma =\left[\begin{array}{c} \gamma_{1}\\ \gamma_{2}\\ \gamma_{3} \end{array}\right]$$

of dimensions 9 × 9 and 9 × 3, have the block-row elements (for *i* = 1,2,3) as follows:

(74) $$\Gamma_{i}=\frac{\partial }{\partial \text{c}}[\Psi (\text{h}+{\text{b}}_{h})\text{T}{\text{e}}_{i}],\gamma_{i}=\frac{\partial }{\partial {\text{b}}_{h}}[\Psi (h+{\text{b}}_{h})\text{T}{\text{e}}_{i}]$$

Stacking together all the previously computed gradients of the Lie derivatives, we form the observability matrix O as:

(75) $$𝒪=\left[\begin{array}{c} \nabla {𝔏}^{0}h_{1}^{*}\\ \nabla {𝔏}^{0}h_{2}^{*}\\ \nabla {𝔏}^{0}h_{3}^{*}\\ \nabla {𝔏}_{{\text{f}}_{0}}^{1}h_{2}^{*}\\ \nabla {𝔏}_{{\text{f}}_{0}}^{1}h_{3}^{*}\\ \nabla {𝔏}_{\underline{{\text{f}}_{1}}}^{1}h_{1}^{*}\\ \nabla {𝔏}^{0}h_{4}^{*} \end{array}\right]=\left[\begin{array}{cccccc} \Psi (h+b_{h}) & 0_{3\times 3} & 0_{3\times 3} & 0_{3\times 3} & 0_{3\times 3} & C_{n}^{b}\\ C_{b}^{c}\Psi (m_{v}^{n}) & 0_{3\times 3} & 0_{3\times 3} & C_{b}^{c}C_{n}^{b} & 0_{3\times 3} & 0_{3\times 3}\\ C_{b}^{c}\Psi (m_{h}^{n}) & 0_{3\times 3} & 0_{3\times 3} & 0_{3\times 3} & C_{b}^{c}C_{n}^{b} & 0_{3\times 3}\\ X_{1} & -C_{b}^{c}\left[C_{n}^{b}m_{v}^{n}\times \right] & C_{b}^{c}C_{n}^{b}\left[l_{v}^{n}\times \right] & C_{b}^{c}\left[b_{g}\times \right]C_{n}^{b} & 0_{3\times 3} & 0_{3\times 3}\\ X_{2} & -C_{b}^{c}\left[C_{n}^{b}m_{h}^{n}\times \right] & C_{b}^{c}C_{n}^{b}\left[l_{h}^{n}\times \right] & 0_{3\times 3} & C_{b}^{c}\left[b_{g}\times \right]C_{n}^{b} & 0_{3\times 3}\\ \Gamma & 0_{9\times 3} & 0_{9\times 3} & 0_{9\times 3} & 0_{9\times 3} & \gamma \\ 2M & 0_{6\times 3} & 0_{6\times 3} & 0_{6\times 3} & 0_{6\times 3} & 0_{6\times 3} \end{array}\right]$$

In order to prove that the system described by Equations (50)–(57) is observable, we show that the matrix O is full rank (*i.e.*, the state space of the system is spanned by the gradients of the Lie derivatives of the measurement functions [16,17]). Before presenting the main result of this section, we first present the following two lemmas whose proofs are detailed in Appendixs B and C, respectively.

Lemma 1: The 15 × 12 matrix:
  (76) $$\text{A}=\left[\begin{array}{cc} \Gamma & \gamma \\ 2\text{M} & 0_{6\times 3} \end{array}\right]$$
  is rank 9 if at least one degree of rotational freedom is excited.
Lemma 2: The 6 × 6 matrix:
  (77) $$B=\left[\begin{array}{cc} -C_{b}^{c}\left[C_{n}^{b}m_{v}^{n}\times \right] & C_{b}^{c}C_{n}^{b}\left[l_{v}^{n}\times \right]\\ -C_{b}^{c}\left[C_{n}^{b}m_{h}^{n}\times \right] & C_{b}^{c}C_{n}^{b}\left[l_{h}^{n}\times \right] \end{array}\right]$$
  is full rank if two linearly independent horizontal lines are observed.

The observability matrix O (*cf.* Equation (75)) is full rank when: (a) at least one degree of rotational freedom is excited; (b) at least one vertical line and two linearly independent horizontal lines are observed. The proofs are given in Appendix D.

## 5. Experimental Results

### 5.1. Hardware Description

In order to demonstrate the validity of the proposed algorithm in realistic situations, we conducted indoor experiments using a testbed that consists of an ISIS IMU, a Firewire camera, a magnetometer, and a computer for data acquisition. The IMU, the camera, and the magnetometer were rigidly mounted on the chassis and their relative pose did not change during the experiment. The magnetometer was placed far from current wires, computer, and ferromagnetic materials. The raw data were delivered through an USB interface to the computer. The intrinsic parameters of the camera and transformation among the IMU, the camera and the magnetometer were calibrated prior to the experiment and were treated as constants. A monocular camera cannot determine the depth information. In order to solve the problem, the height of the camera from the ground plane was measured, and the lines which are on the ground and perpendicular to the ground were chosen as landmarks. We used basic trigonometry to determine the depth information. **m**^c^ can be calculated as measurements. Accuracies of the sensors are listed in Table 1. The IMU, camera and magnetometer were electronically time-synchronized.

### 5.2. Experiment Profile

**Figure 2.**
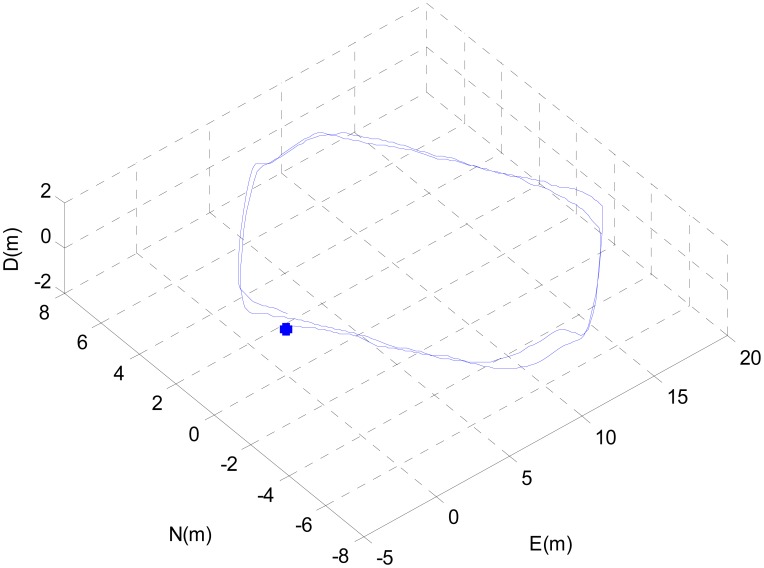
The estimated 3-D trajectory of the pushcart.

A short distance experiment was carried out in the hallway. The testbed was rigidly mounted on the chassis of a pushcart. The estimated 3-D trajectory of the pushcart can be seen in Figure 2. The initial position of the pushcart is denoted by ‘*’.

The line features were extracted using the Hough transform. The line-points can be easily calculated with the detection values (*ρ*,*θ*) from the Hough transform. It was assumed that the camera measurements were corrupted by additive white Gaussian noise with a standard deviation of 2 pixels. The actual pixel noise is less than 2 pixels. However, in order to compensate the existence of the unmodeled nonlinearities and imperfect camera calibration, the noise standard deviation was increased to 2 pixels.

### 5.3. Algorithm Performance

The trajectory of the pushcart included two loops. The final position estimate, expressed with respect to the starting pose, is [0.81,0.59,0.06]*^T^* m. From the initial and final parking spots of the pushcart, it is known that the true final position expressed with respect to the initial pose is approximately [0,0,0]*^T^* m. Thus, the final position error is approximately 1 m at the end of a trajectory of 100 m, *i.e.*, an error of 1% of the travelled distance.

**Figure 3.**
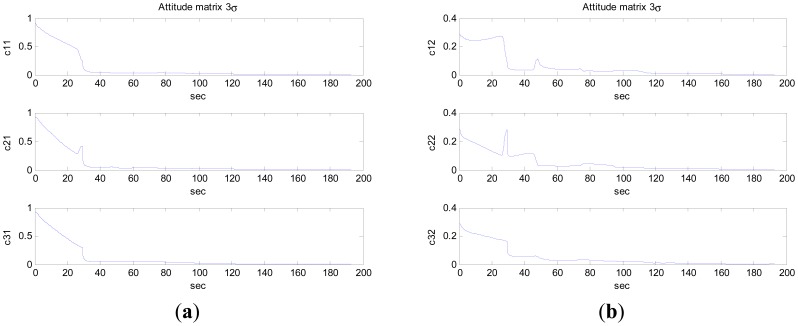

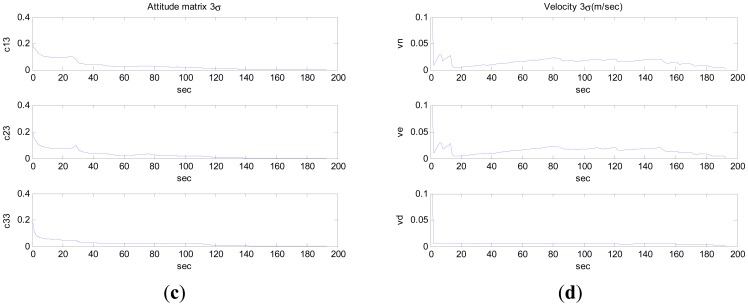
The 3*σ* bounds for the errors in the attitude matrix and the velocities in the *n* frame.

It is noteworthy that the camera motion was almost in parallel to the optical axis, a condition which is particularly adverse for the image-based motion estimation algorithms [18]. In Figure 3, the 3*σ* bounds for the errors in the attitude matrix and the velocities along the three axes are shown. The plotted values are 3-times the square roots of the corresponding diagonal elements of the state covariance matrix. Position errors were mainly caused by the precision of visual measurement and attitude estimate, especially attitude estimate. Because the steel framed buildings affect the magnetic field, the precision of attitude estimate by inertial/magnetic sensors is not high.

## 6. Conclusions

In this paper, we have studied observability of a MKF-based algorithm for indoor navigation using visual/inertial/magnetic sensors. The estimation algorithm uses a compact matrix notation to produce the matrix estimate and the estimation error covariance matrix. The MKF is a natural and straightforward extension of the ordinary KF. Compared with the ordinary KF, the MKF model consists of both pseudo-linear process model and nonlinear measurement model.

The observability of the nonlinear system describing the integrated navigation with visual/inertial/magnetic sensors was investigated by employing the observability rank conditions defined with the Lie derivatives. The pseudo-linear process help simplifying the gradient operator in the process of observability analysis. This paper has for the first time proved that the observability matrix is full rank when: (a) at least one degree of rotational freedom is excited; (b) at least two linearly independent horizontal lines and one vertical line are observed. When these conditions are satisfied, the state matrix that contains the body attitude matrix, the gyro bias vector, relative velocity vector, the dual part of landmark and the magnetic variation superimposed to the magnetic reference vector are observable. The indoor experimental results have demonstrated the correctness of observability conditions.

## Figures and Tables

**Table 1.** Accuracies of the Sensors.

**Sensors**	**Number**	**Accuracies**	**Sampling Rates**
IMU	1	gyro drift: <0.01°/s (short term)	100 Hz
		accelerometer bias: ±2 mg (short term)	
Camera	1	Resolution: 656 × 490	10 Hz
		focus length: 25 mm	
		field of view: 60°	
Magnetometer	1	*σ_bh_*:10 u.a./s(×10^−3^)	100 Hz
		*α*:10 s^−1^	
		*σ_h_*:1 mGauss	

## Appendix A

For arbitrary two vector **p** and **q**

(A1) $$\begin{array}{l} \Psi (\text{p})\Xi (\text{q})\text{c}\\ =[\begin{array}{ccc} {\text{p}}_{1}{\text{I}}_{3} & p_{2}{\text{I}}_{3} & p_{2}{\text{I}}_{3} \end{array}]\left[\begin{array}{ccc} \left[\text{q}\times \right] & 0_{3\times 3} & 0_{3\times 3}\\ 0_{3\times 3} & \left[\text{q}\times \right] & 0_{3\times 3}\\ 0_{3\times 3} & 0_{3\times 3} & \left[\text{q}\times \right] \end{array}\right]\left[\begin{array}{c} {\text{c}}_{1}\\ {\text{c}}_{2}\\ {\text{c}}_{3} \end{array}\right]\\ =p_{1}{\text{I}}_{3}[\text{q}\times ]{\text{c}}_{1}+p_{2}{\text{I}}_{3}[\text{q}\times ]{\text{c}}_{2}+p_{3}{\text{I}}_{3}[\text{q}\times ]{\text{c}}_{3}\\ =[\text{q}\times ]{\text{c}}_{1}p_{1}+[\text{q}\times ]{\text{c}}_{2}p_{2}+[\text{q}\times ]{\text{c}}_{3}p_{3}\\ =\left[\begin{array}{ccc} {}[\text{q}\times ]{\text{c}}_{1} & [\text{q}\times ]{\text{c}}_{1} & [\text{q}\times ]{\text{c}}_{1} \end{array}\right]\left[\begin{array}{c} p_{1}\\ p_{2}\\ p_{3} \end{array}\right]\\ =[\text{q}\times ]{\text{C}}_{n}^{b}p \end{array}$$

## Appendix B

We compute **Γ** and **γ**:

(B1) $$\Gamma =\left[\begin{array}{ccccccccc} 0 & 0 & 0 & 0 & 0 & 0 & 0 & 0 & 0\\ 0 & 0 & h_{1} & 0 & 0 & h_{2} & 0 & 0 & h_{3}\\ 0 & -h_{1} & 0 & 0 & -h_{2} & 0 & 0 & -h_{3} & 0\\ 0 & 0 & -h_{1} & 0 & 0 & -h_{2} & 0 & 0 & -h_{3}\\ 0 & 0 & 0 & 0 & 0 & 0 & 0 & 0 & 0\\ h_{1} & 0 & 0 & h_{2} & 0 & 0 & h_{3} & 0 & 0\\ 0 & h_{1} & 0 & 0 & h_{2} & 0 & 0 & h_{3} & 0\\ -h_{1} & 0 & 0 & -h_{2} & 0 & 0 & -h_{3} & 0 & 0\\ 0 & 0 & 0 & 0 & 0 & 0 & 0 & 0 & 0 \end{array}\right]\begin{array}{c} \begin{array}{c} 1\\ 2\\ 3 \end{array}\}\omega_{x}\\ \begin{array}{c} 4\\ 5\\ 6 \end{array}\}\omega_{y}\\ \begin{array}{c} 7\\ 8\\ 9 \end{array}\}\omega_{z} \end{array}$$

(B2) $$\gamma =\left[\begin{array}{ccc} 0 & 0 & 0\\ c_{31} & c_{32} & c_{33}\\ -c_{21} & -c_{22} & -c_{23}\\ -c_{31} & -c_{32} & -c_{33}\\ 0 & 0 & 0\\ c_{11} & c_{12} & c_{13}\\ c_{21} & c_{22} & c_{23}\\ -c_{11} & -c_{12} & -c_{13}\\ 0 & 0 & 0 \end{array}\right]\begin{array}{c} \begin{array}{c} 1\\ 2\\ 3 \end{array}\}\omega_{x}\\ \begin{array}{c} 4\\ 5\\ 6 \end{array}\}\omega_{y}\\ \begin{array}{c} 7\\ 8\\ 9 \end{array}\}\omega_{z} \end{array}$$

where **h** + **b***_h_* = [*h*_1_ *h*_2_ *h*_3_]*^T^*. The variables, shown on the right side and next to the row numbers indicate the control input that is involved in using the corresponding row numbers: when a row is retained in the observability matrix (e.g., the second row), the underlying assumption is that the corresponding control input (*i.e.*, ω*_x_*) is nonzero.

If none of the components in **h** + **b***_h_* is zero, we can obtain:

(B3) det(A{4,6,7,10:15})≠0

(B4) det(A{2,3,8,10:15})≠0

(B5) det(A{2,3,6,10:15})≠0

It is worth-mentioning that each selection of the rows is based on two nonzero components of $\omega_{ib}^{b}={\left[\omega_{x}\omega_{y}\omega_{z}\right]}^{T}$. For example, we can use either Equation (B3) which assumes that *ω_y_* and *ω_z_* are nonzero, or Equation (B4) which assumes that *ω_x_* and *ω_z_* are nonzero, or Equation (B5) which assumes that *ω_x_* and *ω_y_* are nonzero.

Only if the rotational axis does not coincide with b coordinate axis, will one degree of rotation have two nonzero components of $\omega_{ib}^{b}$. The rank of **A** is 9 if at last one degree of rotational freedom is excited.

## Appendix C

(C1) $$\left|\text{B}\right|=\left|{\text{C}}_{n}^{b}\right|\left|\left[\begin{array}{cc} -\left[{\text{C}}_{n}^{b}{\text{m}}_{v}^{b}\times \right] & {\text{C}}_{n}^{b}\left[{\text{l}}_{v}^{b}\times \right]\\ -\left[{\text{C}}_{n}^{b}{\text{m}}_{h}^{b}\times \right] & {\text{C}}_{n}^{b}\left[{\text{l}}_{h}^{b}\times \right] \end{array}\right]\right|$$

Note that ${\text{C}}_{b}^{c}$ is full rank.

(C2) $$\det(\text{B})=(l_{h2}m_{h1}-l_{h1}m_{h2})(m_{v1}m_{h2}-m_{v2}m_{h1})+{\left(l_{h2}m_{v1}-l_{h1}m_{v2}\right)}^{2}m_{h3}$$

where:

(C3) $${\text{l}}_{v}^{n}={\left[\begin{array}{ccc} 0 & 0 & 1 \end{array}\right]}^{T},{\text{m}}_{v}^{n}={\left[\begin{array}{ccc} m_{v1} & m_{v2} & 0 \end{array}\right]}^{T}$$

(C4) $${\text{l}}_{h}^{n}={\left[\begin{array}{ccc} l_{h1} & l_{h_{2}} & 0 \end{array}\right]}^{T},{\text{m}}_{h}^{n}={\left[\begin{array}{ccc} m_{h1} & m_{h2} & m_{h3} \end{array}\right]}^{T}$$

det(**B**) = 0 when: (a); *m_h_*_1_ = *m_h_*_2_= 0 (b) ${\text{l}}_{h}^{n}$ and ${\text{m}}_{v}^{n}$ are linearly dependent. Only (a) and (b) are both satisfied, **B** is not full rank. To avoid the situation, two linearly independent horizontal lines are observed or the line in the plane (*z^n^* = 0) is not chosen. In practice, the former is more feasible. Even one of horizontal line is linearly dependent with ${\text{m}}_{v}^{n}$, the another is linearly independent with ${\text{m}}_{v}^{n}$. Then, (b) is not satisfied. So **B** is full rank if two linearly independent horizontal lines are observed.

## Appendix D

Step (1)
  Based on Lemma 1, the rank of **A** is 9. We diagonalize it with the Gaussian elimination:
  (D1) $$\left[\begin{array}{cccccc} \Psi (\text{h}+{\text{b}}_{h}) & 0_{3\times 3} & 0_{3\times 3} & 0_{3\times 3} & 0_{3\times 3} & {\text{C}}_{n}^{\text{b}}\\ {\text{C}}_{\text{b}}^{c}\Psi ({\text{m}}_{v}^{n}) & 0_{3\times 3} & 0_{3\times 3} & {\text{C}}_{b}^{c}{\text{C}}_{n}^{b} & 0_{3\times 3} & 0_{3\times 3}\\ {\text{C}}_{b}^{c}\Psi ({\text{m}}_{h}^{n}) & 0_{3\times 3} & 0_{3\times 3} & 0_{3\times 3} & {\text{C}}_{b}^{c}{\text{C}}_{n}^{b} & 0_{3\times 3}\\ {\text{X}}_{1} & -{\text{C}}_{b}^{c}\left[{\text{C}}_{n}^{b}{\text{m}}_{v}^{n}\times \right] & {\text{C}}_{b}^{c}{\text{C}}_{n}^{b}\left[{\text{l}}_{v}^{n}\times \right] & {\text{C}}_{b}^{c}\left[b_{g}\times \right]{\text{C}}_{n}^{b} & 0_{3\times 3} & 0_{3\times 3}\\ {\text{X}}_{2} & -{\text{C}}_{b}^{c}\left[{\text{C}}_{n}^{b}{\text{m}}_{h}^{n}\times \right] & {\text{C}}_{b}^{c}{\text{C}}_{n}^{b}\left[{\text{l}}_{h}^{n}\times \right] & 0_{3\times 3} & {\text{C}}_{b}^{c}\left[b_{g}\times \right]{\text{C}}_{n}^{b} & 0_{3\times 3}\\ {\text{I}}_{9} & 0_{9\times 3} & 0_{9\times 3} & 0_{9\times 3} & 0_{9\times 3} & 0_{9\times 3}\\ 0_{6\times 9} & 0_{6\times 3} & 0_{6\times 3} & 0_{6\times 3} & 0_{6\times 3} & 0_{6\times 3} \end{array}\right]$$
  Then we can easily eliminate all other elements of the first columns:
  (D2) $$\left[\begin{array}{cccccc} 0_{3\times 9} & 0_{3\times 3} & 0_{3\times 3} & 0_{3\times 3} & 0_{3\times 3} & {\text{C}}_{n}^{\text{b}}\\ 0_{3\times 9} & 0_{3\times 3} & 0_{3\times 3} & {\text{C}}_{\text{b}}^{c}{\text{C}}_{n}^{b} & 0_{3\times 3} & 0_{3\times 3}\\ 0_{3\times 9} & 0_{3\times 3} & 0_{3\times 3} & 0_{3\times 3} & {\text{C}}_{b}^{c}{\text{C}}_{n}^{b} & 0_{3\times 3}\\ 0_{3\times 9} & -{\text{C}}_{b}^{c}\left[{\text{C}}_{n}^{b}{\text{m}}_{v}^{n}\times \right] & {\text{C}}_{b}^{c}{\text{C}}_{n}^{b}\left[{\text{l}}_{v}^{n}\times \right] & {\text{C}}_{b}^{c}\left[b_{g}\times \right]{\text{C}}_{n}^{b} & 0_{3\times 3} & 0_{3\times 3}\\ 0_{3\times 9} & -{\text{C}}_{b}^{c}\left[{\text{C}}_{n}^{b}{\text{m}}_{h}^{n}\times \right] & {\text{C}}_{b}^{c}{\text{C}}_{n}^{b}\left[{\text{l}}_{h}^{n}\times \right] & 0_{3\times 3} & {\text{C}}_{b}^{c}\left[b_{g}\times \right]{\text{C}}_{n}^{b} & 0_{3\times 3}\\ {\text{I}}_{9} & 0_{9\times 3} & 0_{9\times 3} & 0_{9\times 3} & 0_{9\times 3} & 0_{9\times 3}\\ 0_{6\times 9} & 0_{6\times 3} & 0_{6\times 3} & 0_{6\times 3} & 0_{6\times 3} & 0_{6\times 3} \end{array}\right]$$
Step (2)
  We can eliminate all other elements of the fourth and fifth columns using ${\text{C}}_{b}^{c}{\text{C}}_{n}^{b}$, respectively:
  (D3) $$\left[\begin{array}{cccccc} 0_{3\times 9} & 0_{3\times 3} & 0_{3\times 3} & 0_{3\times 3} & 0_{3\times 3} & {\text{C}}_{n}^{b}\\ 0_{3\times 9} & 0_{3\times 3} & 0_{3\times 3} & {\text{C}}_{b}^{c}{\text{C}}_{n}^{b} & 0_{3\times 3} & 0_{3\times 3}\\ 0_{3\times 9} & 0_{3\times 3} & 0_{3\times 3} & 0_{3\times 3} & {\text{C}}_{b}^{c}{\text{C}}_{n}^{b} & 0_{3\times 3}\\ 0_{3\times 9} & -{\text{C}}_{b}^{c}\left[{\text{C}}_{n}^{b}{\text{m}}_{v}^{n}\times \right] & {\text{C}}_{b}^{c}{\text{C}}_{n}^{b}\left[{\text{l}}_{v}^{n}\times \right] & 0_{3\times 3} & 0_{3\times 3} & 0_{3\times 3}\\ 0_{3\times 9} & -{\text{C}}_{b}^{c}\left[{\text{C}}_{n}^{b}{\text{m}}_{h}^{n}\times \right] & {\text{C}}_{b}^{c}{\text{C}}_{n}^{b}\left[{\text{l}}_{h}^{n}\times \right] & 0_{3\times 3} & 0_{3\times 3} & 0_{3\times 3}\\ {\text{I}}_{9} & 0_{9\times 3} & 0_{9\times 3} & 0_{9\times 3} & 0_{9\times 3} & 0_{9\times 3}\\ 0_{6\times 9} & 0_{6\times 3} & 0_{6\times 3} & 0_{6\times 3} & 0_{6\times 3} & 0_{6\times 3} \end{array}\right]$$
Step (3)
  Based on Lemma 2, **B** is full rank, thus we diagonalize it with the Gaussian elimination:
  (D4) $$\left[\begin{array}{cccccc} 0_{3\times 9} & 0_{3\times 3} & 0_{3\times 3} & 0_{3\times 3} & 0_{3\times 3} & {\text{C}}_{n}^{b}\\ 0_{3\times 9} & 0_{3\times 3} & 0_{3\times 3} & {\text{C}}_{b}^{c}{\text{C}}_{n}^{b} & 0_{3\times 3} & 0_{3\times 3}\\ 0_{3\times 9} & 0_{3\times 3} & 0_{3\times 3} & 0_{3\times 3} & {\text{C}}_{b}^{c}{\text{C}}_{n}^{b} & 0_{3\times 3}\\ 0_{3\times 9} & {\text{I}}_{3} & 0_{3\times 3} & 0_{3\times 3} & 0_{3\times 3} & 0_{3\times 3}\\ 0_{3\times 9} & 0_{3\times 3} & {\text{I}}_{3} & 0_{3\times 3} & 0_{3\times 3} & 0_{3\times 3}\\ {\text{I}}_{9} & 0_{9\times 3} & 0_{9\times 3} & 0_{9\times 3} & 0_{9\times 3} & 0_{9\times 3}\\ 0_{6\times 3} & 0_{6\times 3} & 0_{6\times 3} & 0_{6\times 3} & 0_{6\times 3} & 0_{6\times 3} \end{array}\right]$$
  Note that ${\text{C}}_{b}^{c}{\text{C}}_{n}^{b}$ and ${\text{C}}_{n}^{b}$ are both full rank. It is easy to see the matrix is full rank, indicating that the matrix O is also full rank.
